# Supplementary material for: Home Range, Movement, and Nest Use of Hedgehogs (Erinaceus europaeus) in an Urban Environment Prior to Hibernation
Source: Animals (Basel). 2023 Dec 29;14(1):130. doi: 10.3390/ani14010130 (PMC10778517; doi:10.3390/ani14010130)
Supplement: Supplementary file 1 [file animals-14-00130-s001.zip › animals-2752629-supplementary.pdf]

Supplementary material

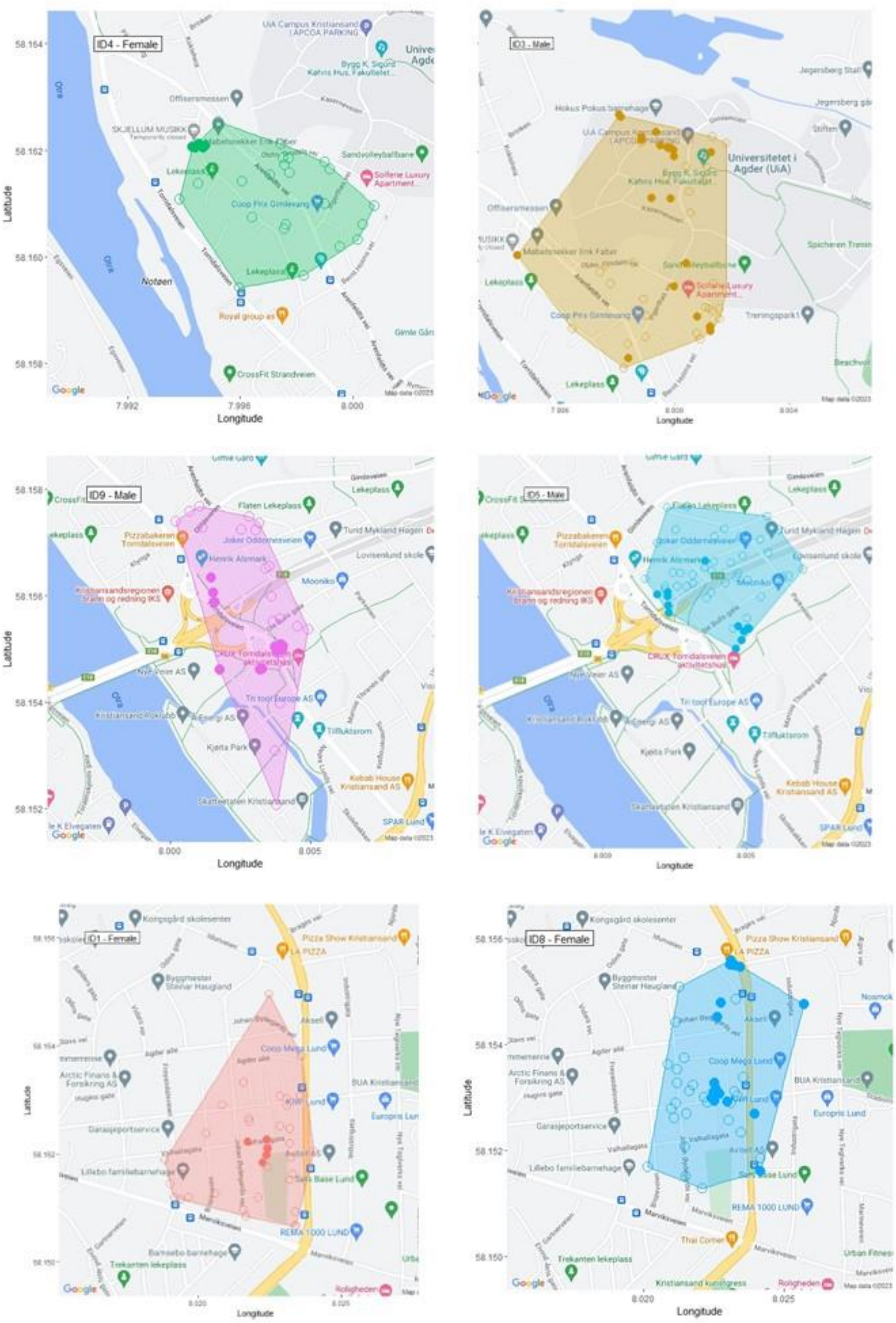

Figure S1. 100% MCP home ranges of all six individuals. Open symbols represent localization where the animal was active (during night) and closed symbols represent nest sites. Due to GPS-inaccuracy multiple nest sites positioned close together represent a nest with several localization, but at exactly the same place.

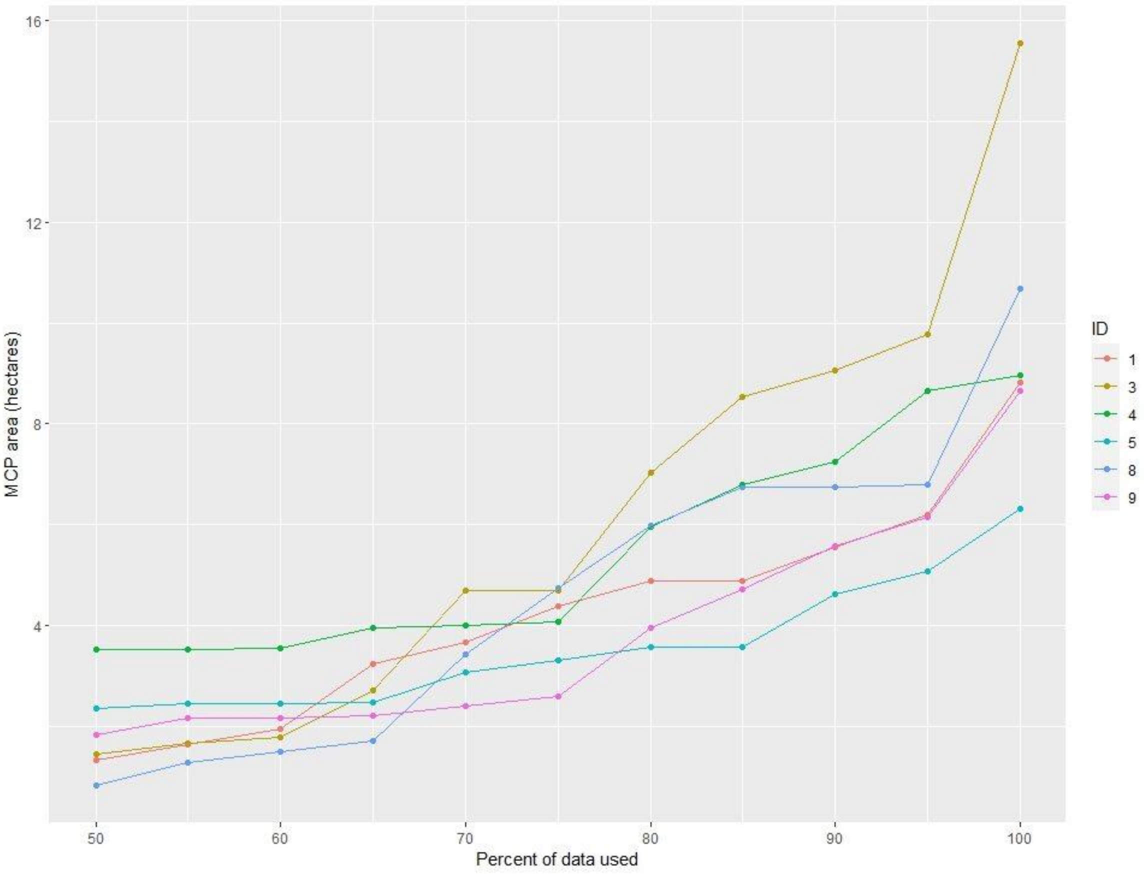

Figure S2. Home range asymptote figure, with maximum convex polygon (MCP) area as a function of percentage of data used for each individual hedgehog. The shape of the curves suggest that our home range estimates are underestimated, as the asymptotes still rise at 100% MCPs.
